# Supplementary material for: The interactome of intact mitochondria by cross-linking mass spectrometry provides evidence for coexisting respiratory supercomplexes
Source: Mol Cell Proteomics. 2017 Dec 8;17(2):216–32. doi: 10.1074/mcp.RA117.000470 (PMC5795388; doi:10.1074/mcp.RA117.000470)
Supplement: Supplemental Data [file supp_RA117.000470_133922_0_supp_23978_fzffwf.zip › spectra_annotation/mito_spectra_annotation/302-1-2-1-2-1.pdf]

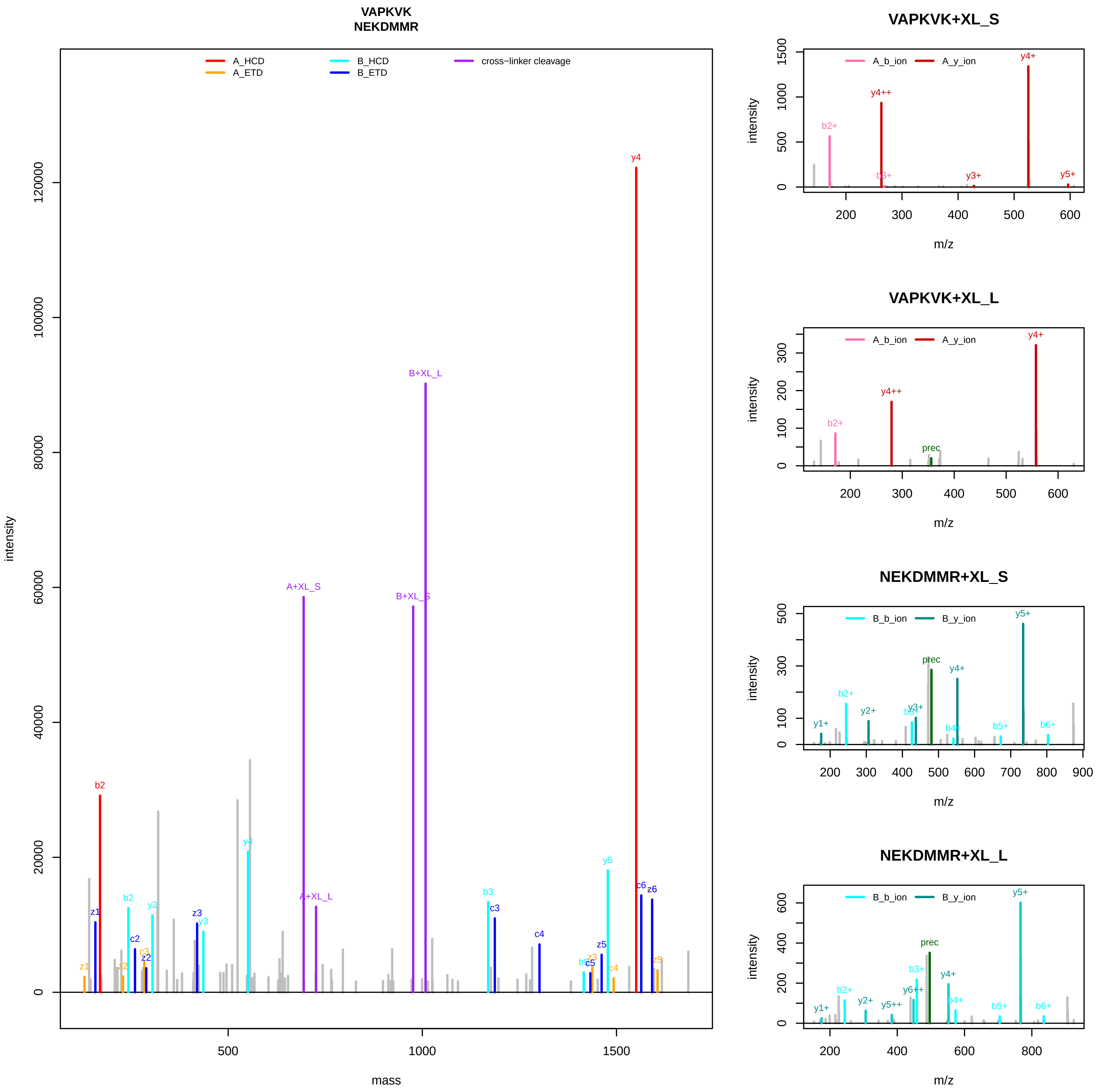

VAPKVK
NEKDMMR
A_HCD
A_ETD
B_HCD
B_ETD
cross−linker cleavage
intensity
mass
y4
B+XL_L
A+XL_S
B+XL_S
A+XL_L
b2
z1
c2
z2
c3
y2
z3
y3
b3
c3
c4
y5
c5
z5
c6
z6
c4
z5
VAPKVK+XL_S
A_b_ion
A_y_ion
y4+
y4++
b2+
b3+
y3+
y5+
m/z
VAPKVK+XL_L
prec
NEKDMMR+XL_S
B_b_ion
B_y_ion
y5+
y4+
y3+
y2+
y1+
b5+
b6+
NEKDMMR+XL_L
y6++
y5++
